# Physical and Emotional Health Problems Experienced by Youth Engaged in Physical Fighting and Weapon Carrying

**DOI:** 10.1371/journal.pone.0056403

**Published:** 2013-02-21

**Authors:** Sophie D. Walsh, Michal Molcho, Wendy Craig, Yossi Harel-Fisch, Quynh Huynh, Atif Kukaswadia, Katrin Aasvee, Dora Várnai, Veronika Ottova, Ulrike Ravens-Sieberer, William Pickett

**Affiliations:** 1 Department of Criminology, Bar Ilan University, Ramat Gan, Israel; 2 School of Health Science, National University of Ireland, Galway, Ireland; 3 Department of Psychology, Queen’s University, Kingston, Ontario, Canada; 4 School of Education, Bar Ilan University, Ramat Gan, Israel; 5 Department of Community Health and Epidemiology, Queen’s University, Kingston, Ontario, Canada; 6 Social Program Evaluation Group, Queen’s University, Kingston, Ontario, Canada; 7 Department of Chronic Diseases, National Institute for Health Development, Tallinn, Estonia; 8 National Institute of Child Health, Budapest, Hungary; 9 Research Unit Child Public Health, University Medical Center Hamburg-Eppendorf, Hamburg-Eppendorf, Germany; The University of South Wales, Australia

## Abstract

Then aims of the current study were 1) to provide cross-national estimates of the prevalence of physical fighting and weapon carrying among adolescents aged 11–15 years; (2) To examine the possible effects of physical fighting and weapon carrying on the occurrence of physical (medically treated injuries) and emotional health outcomes (multiple health complaints) among adolescents within the theoretical framework of Problem Behaviour Theory. 20,125 adolescents aged 11–15 in five countries (Belgium, Israel, USA, Canada, FYR Macedonia) were surveyed via the 2006 Health Behaviour in School Aged Children survey. Prevalence was calculated for physical fighting and weapon carrying along with physical and emotional measures that potentially result from violence. Regression analyses were used to quantify associations between violence/weapon carrying and the potential health consequences within each country. Large variations in fighting and weapon carrying were observed across countries. Boys reported more frequent episodes of fighting/weapon carrying and medically attended injuries in every country, while girls reported more emotional symptoms. Although there were some notable variations in findings between different participating countries, increased weapon carrying and physical fighting were both independently and consistently associated with more frequent reports of the potential health outcomes. Adolescents engaging in fighting and weapon carrying are also at risk for physical and emotional health outcomes. Involvement in fighting and weapon carrying can be seen as part of a constellation of risk behaviours with obvious health implications. Our findings also highlight the importance of the cultural context when examining the nature of violent behaviour for adolescents.

## Introduction

Youth violence is a global health issue of high public health importance [1], [2], [3], [4]. On the one hand, encouraging recent trend analysis from 2002–2010 in 30 countries in Europe, North American and the Middle East [5] suggests that in a majority of countries (63%) rates of physical fighting among 11–15 year old adolescents have decreased. However, despite overall decreasing levels, figures also show wide variability in the frequency of fighting across countries [6] and consistent correlations between youth violence and negative health outcomes such as substance use, depression, involvement with deviant peers and antisocial tendencies [7], [8]. Figures suggest a substantial number of youth still involved in youth violence. For example, according to recent data in the US [7], 30.9% of youth reported being in a physical fight during the past 12 months and 14.1% reported carrying a gun, knife, club, or similar weapon on their person in the past 30 days. Current data from Israel [9] show 50.6% of adolescent boys and 13.9% of girls reported taking part in a physical fight in the previous 12 months and 17.4% boys and 3.8% girls carried a weapon to school in the past 30 days. The substantial numbers and the associated health correlates demand further understanding of the phenomena of youth violence around the globe.

The current paper focuses on physical fighting [10] as an indicator of violence and weapon carrying [11] as a risk factor for violence. These behaviours are potential sentinel indicators of a problem behaviour lifestyle with multiple consequences, both physical [12] and emotional [13]. It must be remembered that weapon carrying in and of itself may not be an indicator of violence as young people may be carrying weapons for self defense. As such we term it a risk factor for violence and in the current paper we control for reported victimization from bullying in order to limit the cases in which weapon carrying is for purposes of defense. Both physical fighting and weapon carrying are highly correlated with use of illicit substances [14], [15] and early involvement in sexual behaviours [16]. They are also associated with a myriad of health-related outcomes and other forms of violence including bullying [13], [16], poor academic achievement and reduced engagement in school activities [17], suicidal ideation and behaviour [18], [19], and other measures of emotional distress [20]. At a more extreme level, adolescents with access to handguns are also more likely to report risk behaviours and past injury (Loh, Walton, Harrison t al., 2010). While it is clear that both physical fighting and weapon carrying are important indicators of youth violence, few international comparisons have been carried out of the effects of these behaviours on the health of youth populations. Such analyses are useful for international surveillance efforts, and they have considerable potential for the testing of common social theories.

The theoretical framework guiding this research is Problem Behaviour Theory [21], [22], [23], in which risk behaviors are considered in a psychosocial framework which emphasizes both the costs and benefits of risk behaviors for adolescents. Problem Behavior Theory [22], [24], [25] holds a covariate perspective in which risk behaviors exist in an organized constellation and are inter-related and strongly correlated. According to Problem Theory, problem behaviours essentially represent a constellation of symptoms for a troubled adolescent, and together they contribute to a trajectory of poor health outcomes. Empirical evidence for the problem behaviour perspective [26] has suggested that covariance of risk behaviours is particular evident with problem behaviours (e.g., drug use, alcohol, delinquency and sexual precocity) and characteristic of deviance prone youngsters. Problem Behaviour theory has been a useful framework for examining similarities and differences in relationships between risk behaviours across various countries [27], [28] and although there has been little research specifically examining the relevance of Problem Behaviour theory to the arena of peer violence, it has been used to understand a problem behavior syndrome, operationalized by vandalism, general deviance, school misconduct, theft, and assault measures across 8 countries [29] and to explain the findings that youth engaged in fighting have been shown to have higher levels of suicidal ideation, weapon carrying, using cocaine and driving while intoxicated [10]. As such, Problem Behaviour Theory would suggest that engagement in fighting or weapon carrying would be part of a lifestyle of problematic behaviours and, as such, will increase the risk of a variety of adverse health effects.

Current literature emphasizes the role of context as a determinant of violence [30] and the importance of examining context on a number of geographic and cultural levels [3]. World Health Organization estimates suggest that countries exhibit a range of policies, levels of violence, and rates of unintentional injury [31], [32], [33]. Where data from multiple countries exist, this permits a unique opportunity to examine the prevalence of violence and its association with mental and physical health across diverse contexts. The current study involves adolescents from five countries: Belgium (French speaking), Canada, Israel, FYR Macedonia and the USA. These are five countries which represent a diversity not only in recent estimates of violence but also in political and social context, policies concerning weapon carrying, levels of societal violence and intervention strategies. In terms of estimates of youth violence, according to figures from the 2005–6 Health Behaviors of School Aged Children (HBSC-WHO) survey, these countries represent a diverse range of frequencies of youth violence. From among the 41 countries surveyed as to the percentage of 15 year olds involved in at least three incidences of physical fighting in the past 12 months, adolescents from French speaking Belgium reported the highest frequencies (ranked 1), Israeli adolescents reporting second to lowest frequencies (ranked 40), with adolescents from FYR Macedonia (ranked 6), Canada (ranked 22) and the USA (ranked 32) reporting mid-high, mid and mid-low frequencies. As such, the study represents the opportunity to examine correlates of youth violence in countries with differing levels of violence.

The five countries all differ in terms of social and political context and the study represents adolescents growing up in very different realities. Israel is a relatively young country in which its adolescents are growing up experiencing events of armed conflict [34] and in which compulsory military service for all 18 year olds makes weapon carrying routine in an army context. Since 1991, FYR Macedonia has dealt with challenges of post communist political and economic transition and has seen political unrest, high levels of unemployment and interethnic violence in the lifetimes of the adolescents in this study [35] before entering into the European Union in 2005. While the USA and Canada show many similar values, violent crime and juvenile incarceration rates are notably higher in the USA [36] with high prevalence rates of adolescents carrying guns [37]. Yet, the United States has improved in the prevalence of bullying problems, perhaps connected to a national bullying prevention campaign launched by the U.S. government [38]. According to data from the World Health Organization, Belgium has almost twice the frequency of mortality from youth violence than the European Union average and yet despite having national policies for injury and violence prevention has been highlighted for its low levels of intervention effectiveness [39].

The goal of the present study was to conduct a cross-national analysis of relationships between physical fighting and weapon carrying and two health outcomes: (1) medically treated injuries, as these have been found to be highly associated with an organized set of risk behaviours [21], yet have received little attention as specific outcomes of violence [40], [41]; (2) emotional health outcomes, as the influence of violence on emotional health is appreciated but rarely quantified [1]. Study findings contribute new knowledge surrounding the burden of violence on the health of adolescent populations, as well as to a better theoretical understanding of the effects of violence on the physical and emotional health of populations of young people.

## Methods

### Human Subjects

Each participating country obtained approval to conduct the survey from their respective institutional ethics review board or equivalent regulatory body. Specifically, in Canada, approval was obtained from the General Research Ethics Board, Queen's University and the Public Health Agency of Canada/Health Canada Research Ethics Board and written consent was given by parents. In FYR Macedonia, approval was obtained from the Ministry of Education as well as written parents consent. Ethical approval was obtained from the School Boards of the French-speaking Community of Belgium of the participating schools who exempted parental consent. Participation of children was voluntary. In Israel, approval was obtained from the Ethics Committee of Bar Ilan University and the Chief Scientists Office of the Ministry of education, who gave an exemption for parental consent due to the anonymity and low sensitivity level of the survey. In the US, the study protocol was approved by the Institutional Review Board of the Eunice Kennedy Shriver National Institute of Child Health and Human Development Active consent (signed consent forms) or passive consent (parents could decline to participate) was obtained from parents of participants depending on the rules of the participating school district. In all countries children's participation was voluntary and oral consent was given.

### Study Population and Procedures

We analyzed international data from the 2006 World Health Organization Health Behaviour in School-aged children Survey (WHO-HBSC) [42]. HBSC is a school-based survey of adolescent health behaviours and their underlying determinants, carried out every 4 years simultaneously in participating countries, using an international standardized methodological protocol [43]. The study base includes school children aged 11, 13 and 15 (6^th^, 8^th^ and 10^th^ grade students) in 40 countries in Europe and North America. According to the study protocol, data from each country are gathered from nationally representative samples that include at least 1,500 sampled children in each of the three age groups, with national sample sizes of 4 to 6 thousand students per country. HBSC follows a multi-stage, cluster randomized sampling strategy. Some countries (e.g. Canada, Israel) use a weighting system to account for over-sampling of certain groups but in most countries they are clustered random samples. As such, it is possible to provide estimates and confidence intervals for prevalence that are accurate. The sampling method is based on single classrooms as the sampling unit, where all students belonging to a sampled classroom being included. The HBSC uses a standard, self-administered in-class questionnaire that includes both mandatory and optional items. Further details surrounding methodology of the HBSC can be found elsewhere [43], [44]. In the current study a total of 20,125 children were included: 2492 from French speaking Belgium, 5746 from Canada, 4235 from Israel, 5086 from the FYR Macedonia, and 2566 from the USA.

### Key Measures

The 2006 international survey contained mandatory questions about health behaviours, health status and outcomes, and demographic characteristics. These were asked of all survey participants and included core questions on physical fighting, medically treated injuries, and emotional health. Additional optional items on weapon carrying were assessed in five countries only (Belgium, Israel, USA, Canada, Macedonia), and these countries formed the basis for the present analysis. Response rates at the individual student level ranged from 74% to almost 100% by country.

### Measures of Physical Fighting and Weapon Carrying

#### Physical fighting

Participants were asked, "During the past 12 months how many times were you involved in a physical fight (1- *I have not been in a physical fight* through 5- *four or more times*). Frequency of fighting is a validated construct with extensive use in American and other youth risk behaviour surveys [45]. Reports of *3 or more fights* during the past 12 months were classified as *frequent physical fighting* [42]. Reports of *1–2 fights* during the past 12 months were classified as *infrequent fighting.* A third group comprised those children who did not report any fighting during the past 12 months.

#### Weapon carrying

Participants were asked "During the past 30 days, on how many days did you carry a weapon, such as a gun, knife or club?" (1-*I did not carry a weapon during the past 30 days* through 5–*6 or more days*). The measure of weapon carrying is also considered to be reliable, and is a measure used in most major studies of youth violent behaviours in the Western world [45]. Participants were then classified into those who had and those who had not carried a weapon.

### Other Health Measures

#### Medically treated injury

Participants were asked, "During the past 12 months, how many times were you injured and had to be treated by a doctor or a nurse?" (1- *I was not injured in the past 12 months* through 5- *four or more times*). Wording of these questions and the response categories were based upon research developments in the United States [45], [46] and responses obtained during previous WHO-HBSC surveys [47].

### Emotional Health Outcomes

An established 8-item emotional health symptom scale with excellent psychometric properties was administered to each student [48]. The HBSC symptom checklist has been used in all previous HBSC surveys. The scale represents a non-clinical measure of mental health. Previous research [49], [50] suggests that the scale reflects two dimensions - one psychological and one somatic factor. The scale is flexible in that statistical analyses are meaningful both on single-item level [51] and on sumscore level (Haugland et al., 2001). Participants were asked "In the last six months, how often have you had the following: *Headache, Stomach-ache, Back ache, Feeling low, Irritability or bad temper, Feeling nervous, Difficulties in getting to sleep, feeling dizzy*" (1-*rarely or never* through 5- *about every day*) An outcome of poor emotional health was operationally defined using standard cut-offs (>*about every week*) for at least 2 of the 8 symptoms.

### Statistical Analysis

Analyses were conducted in two stages. In stage 1, descriptive analyses were performed by country to characterize the youth populations in terms of the prevalence of frequent physical fighting and weapon carrying. We also described these populations demographically and by the two health outcomes: medically treated injury and emotional health symptoms. To account for the clustered sampling procedures, standard error estimates were inflated using a conservative design effect of 1.2 as suggested by Roberts et al. [52].

In stage 2, we evaluated associations between physical fighting and weapon carrying and reports of injury and emotional symptoms. The analysis examined: 1) direct effects between indicators of fighting and weapon carrying and the health outcomes; and 2) the consistency of any observed effects across countries. Multi-level regression models with a logit link function were employed, with individual responses by students (level 1) nested within schools (level 2), with this method accounting for clustering at the school level. All analyses were repeated in each of the five countries. Based upon past WHO-HBSC analyses involving backwards elimination processes [12], all models adjusted for: age, gender, socio-economic status (measured using Family Affluence Scores or FAS [44]), physical inactivity, drunkenness, current smoking, and excess time spent with friends. Each covariate item is described extensively, with information about their origins and validity/reliability, in the 2006 international WHO-HBSC protocol. In addition to established covariates, victimization due to bullying was included in all models as a general indication of the perceived need to protect oneself (and hence perhaps carry a weapon) due to past experience as a victim of aggression. All statistical modelling was conducted using SAS (Version 9.1.3, SAS Institute, Cary, NC).

National samples were sufficient to provide confidence intervals of +/−3% for prevalence estimates. Sample sizes from eligible countries that asked about the items of interests ranged from 2492 to 5746, with an approximately equal gender split. Based upon: (1) the observed variability of violence exposures and potential health outcomes, (2) a conservative intra-class correlation of 0.10, (3) two-sided tests with significance level α  = 0.05, these sample sizes provide over 80% power to detect an odds ratio of 1.5 for the groups with highest levels of exposure relative to those with no reported exposures to violence, by gender and within each country.

## Results

20,125 children from Belgium, Canada, Macedonia, Israel and the USA participated in the 2005/2006 WHO-HBSC survey and answered the mandatory and optional items of interest here. Demographic characteristics of these children are profiled by country in Table 1.

**Table 1:** Demographic characteristics of study samples in five HBSC countries, 2006.

Variables	*Belgium-French*	*Canada*	*Israel*	*FYR Macedonia*	*United States*	*Overall*
Total N	2492	5746	4235	5086	2566	20 125
Male %	52.0	47.0	48.9	49.5	47.1	46.6
Mean Age (SD)	14.5 (1.0)	13.8 (1.5)	14.1 (1.6)	13.6 (1.6)	14.4 (1.1)	14.0 (1.5)

Involvement of boys in frequent physical fighting during the previous year ranged from 13.1% in the United States to 26.2% in Belgium-French, with a median of 18.8% (Table 2). Among girls, the prevalence of frequent physical fighting ranged from 3.3% in Israel to 12.7% in Belgium-French, with a median of 6.2%. Involvement in weapon carrying in the last 30 days ranged from 11.3% (Belgium-French) to 22.2% (USA) of boys, and 1.6% (Belgium-French) to 7.1% (USA) of girls.

**Table 2:** Prevalence of physical fighting, weapon carrying, and potential adolescent health outcomes, percentages (standard errors) by gender and country, 2006.

Variables	*Belgium-French* *% (SE)*		*Canada* *% (SE)*		*Israel* *% (SE)*		*FYR Macedonia* *% (SE)*		*United States* *% (SE)*	
	*Boys* *(N = 1 295)*	*Girls* *(N = 1 197)*	*Boys* *(N = 2 698)*	*Girls* *(N = 3 048)*	*Boys* *(N = 1 648)*	*Girls* *(N = 2 587)*	*Boys* *(N = 2 520)*	*Girls* *(N = 2 566)*	*Boys* *(N = 1 208)*	*Girls* *(N = 1 358)*
Physical Fighting (last 12 months):										
*Never*	36.6 (1.3)	66.2 (1.4)	46.7 (1.0)	69.3 (0.8)	45.3 (1.2)	84.3 (0.7)	48.7 (1.0)	75.4 (0.9)	54.5 (1.4)	74.6 (1.2)
*Infrequent (1–2 times)*	36.8 (1.3)	21.1 (1.2)	34.5 (0.9)	20.7 (0.7)	35.5 (1.2)	12.3 (0.6)	32.5 (0.9)	18.5 (0.8)	32.5 (1.3)	19.3 (1.1)
*Frequent (3 or more times)*	26.2 (1.2	12.7 (1.0)	18.8 (0.8)	10.0 (0.5)	19.2 (1.0)	3.3 (0.4)	18.8 (0.8)	6.2 (0.5)	13.1 (1.0)	6.1 (0.7)
Weapon Carrying (last 30 days)	11.3 (0.9)	1.6 (0.4)	16.2 (0.7)	3.5 (0.3)	18.4 (1.0)	3.2 (0.3)	11.9 (0.6)	1.7 (0.3)	22.2 (1.2)	7.1 (0.7)
Both Violent Behaviors	7.1 (0.7)	0.6 (0.2)	6.6 (0.5)	1.3 (0.2)	8.3 (0.7)	1.0 (0.2)	5.2 (0.4)	0.5 (0.1)	6.3 (0.7)	2.1 (0.4)
Medically Attended Injury	49.3 (1.4)	34.8 (1.4)	46.5 (1.0)	37.4 (0.9)	59.8 (1.2)	44.5 (1.0)	33.2 (0.9)	21.3 (0.8)	52.9 (1.4)	41.4 (1.3)
Emotional Health Outcomes	28.3 (1.3)	43.5 (1.4)	26.2 (0.8)	36.5 (0.9)	45.1 (1.2)	53.5 (1.0)	26.4 (0.9)	37.9 (1.0)	32.4 (1.3)	50.6 (1.4)

Prevalence values for children who reported both frequent fighting in the past 12 months, and any weapon carrying in the past 30 days, varied by country and gender (Table 2). Percentages of boys reporting both behaviours varied from 5.2% (FYR Macedonia) to 8.3% (Israel). Among girls, those who reported both behaviours ranged from 0.5% in FYR Macedonia to 2.1% in the United States. In all countries boys reported more weapon carrying and more frequent physical fighting than girls.

Among boys, medically attended injury was more prevalent (33.2% FYR Macedonia to 59.8% Israel) than emotional health problems (26.2% Canada to 45.1% Israel). The prevalence of injury was lower among girls (21.3% FYR Macedonia to 44.5% Israel) when compared with boys. Conversely, emotional health outcomes were reported more often than medical injuries among girls (36.5% Canada to 53.5% Israel) in 4 out of 5 countries compared to boys. Girls also reported more severe emotional problems than boys in all 5 countries. For both genders, levels of injuries and emotional health problems were higher in Israel and the US than in the other three countries.

### Relationships between Violence and Health Outcomes

Strong positive and consistent associations, across all 5 countries, were reported between engagement in fighting and weapon carrying and medically treated injuries and emotional health outcomes Results are presented separately for boys (Table 3) and girls (Table 4).

**Table 3:** Results of logistic regression analysis conducted in five countries: relative odds of two health outcomes associated with (1) physical fighting and (2) weapon carrying, 2006 *(Boys)* *.

Variables	*Belgium-French* *OR (95% CI)*	*Canada* *OR (95% CI)*	*Israel* *OR (95% CI)*	*FYR Macedonia* *OR (95% CI)*	*United States* *OR (95% CI)*
**BOYS**					
***Medically Attended Injury***	*(N = 1295)*	*(N = 2698)*	*(N = 1648)*	*(N = 2520)*	*(N = 1208)*
Physical Fighting (last 12 months):					
*Never*	1.00	1.00	1.00	1.00	1.00
*Infrequent (1–2 times)*	1.73 (1.32–2.28)	1.71 (1.42–2.06)	2.14 (1.67–2.74)	1.96 (1.60–2.38)	1.79 (1.37–2.33)
*Frequent (3 or more times)*	1.85 (1.34–2.55)	2.03 (1.60–2.57)	2.87 (2.05–4.03)	2.33 (1.84–2.96)	1.93 (1.31–2.85)
Weapon Carrying (last 30 days)	1.55 (1.03–2.34)	1.48 (1.18–1.87)	1.55 (1.13–2.13)	2.54 (1.95–3.30)	1.28 (0.95–1.71)
***Emotional Health Outcomes***					
Physical Fighting (last 12 months):					
*Never*	1.00	1.00	1.00	1.00	1.00
*Infrequent (1–2 times)*	1.23 (0.88–1.71)	1.51 (1.21–1.88)	1.33 (1.04–1.71)	1.45 (1.17–1.80)	1.48 (1.11–1.98)
*Frequent (3 or more times)*	2.25 (1.57–3.23)	2.51 (1.93–3.27)	1.96 (1.43–2.70)	2.19 (1.71–2.81)	2.36 (1.51–3.36)
Weapon Carrying (last 30 days)	1.95 (1.28–2.97)	1.96 (1.53–2.51)	2.15 (1.58–2.91)	1.93 (1.47–2.52)	1.63 (1.20–2.20)

* (1) Models simultaneously adjusted for age, socio-economic status, physical activity, smoking, drinking, evenings out with friends, and bullying victimization.

**Table 4:** Results of logistic regression analysis conducted in five countries: relative odds of two health outcomes associated with (1) physical fighting and (2) weapon carrying, 2006 *(Girls)* *.

Variables	*Belgium-French* *OR (95% CI)*	*Canada* *OR (95% CI)*	*Israel* *OR (95% CI)*	*FYR Macedonia* *OR (95% CI)*	*United States* *OR (95% CI)*
**GIRLS**					
***Medically Attended Injury***	*(N = 1197)*	*(N = 3048)*	*(N = 2587)*	*(N = 2566)*	*(N = 1358)*
Physical Fighting (last 12 months):					
*Never*	1.00	1.00	1.00	1.00	1.00
*Infrequent (1–2 times)*	1.80 (1.30–2.47)	1.79 (1.47–2.17)	1.99 (1.53–2.60)	2.39 (1.89–3.02)	1.64 (1.23–2.19)
*Frequent (3 or more times)*	1.93 (1.29–2.87)	2.06 (1.57–2.70)	2.46 (1.45–4.17)	1.99 (1.38–2.89)	1.61 (1.00–2.60)
Weapon Carrying (last 30 days)	1.49 (0.50–4.42)	1.55 (0.99–2.43)	1.79 (1.07–3.00)	4.31 (2.24–8.32)	1.15 (0.73–1.81)
***Emotional Health Outcomes***					
Physical Fighting (last 12 months):					
*Never*	1.00	1.00	1.00	1.00	1.00
*Infrequent (1–2 times)*	1.77 (1.29–2.43)	1.66 (1.36–2.04)	1.63 (1.24–2.14)	1.62 (1.30–2.02)	1.52 (1.13–2.03)
*Frequent (3 or more times)*	2.34 (1.56–3.52)	2.48 (1.88–3.28)	2.96 (1.61–5.43)	2.15 (1.51–3.07)	2.49 (1.48–4.21)
Weapon Carrying (last 30 days)	1.51 (0.45–5.00)	2.49 (1.53–4.05)	1.67 (0.95–2.93)	1.91 (0.96–3.80)	2.61 (1.55–4.39)

* (1) Models simultaneously adjusted for age, socio-economic status, physical activity, smoking, drinking, evenings out with friends, and bullying victimization.

Among boys, there was a remarkable consistency in the strength, dose-dependent nature and significance of these effects observed across countries. The relative odds of medically treated injury for the frequent *vs.* none categories of physical fighting ranged from 1.85 (Belgium-French) to 2.87 (Israel), with a clear graded increase in the odds ratios seen in all five countries from the lowest to most highly frequent levels of exposure (Table 3). Similarly, in four of the five countries a modest and statistically significant increase was observed in the relative odds of injury associated with reports of weapon carrying. In addition, there was an effect in the same direction (albeit not significant) for the one country of exception (United States). These same general patterns were observed for girls, although the estimated increases in odds ratios were not statistically significant in three countries (Belgium-French, Israel, and the United States) for the weapon carrying measures.

For the poor emotional health outcome, in all countries, consistent and statistically significant increases in odds ratios were observed in association with physical fighting and emotional health among boys (Table 3) and girls (Table 4). However, for the weapon carrying measure, while such an association existed in all five countries for boys (Table 3), it was only apparent in the two North American countries (Canada and the United States) among girls.

## Discussion and Conclusions

This large cross-national analysis represents a unique contribution to the study of violence in youth populations. First, it provides contemporary estimates of engagement in physical fighting and weapon carrying among young adolescent children in five countries. Second, it explores relationships between physical fighting and weapon carrying and physical (injury) and emotional health outcomes within the framework of Problem Behaviour Theory (Jessor & Jessor, 1977). Third, it highlights the role of geographical and cultural context by examining the consistency of associations between violence and the health of young people across five diverse countries.

Large variations in fighting and weapon carrying were observed across the five countries, with a consistent gender disparity indicating more frequent involvement among boys, as per the findings of others [1], [53]. While interesting, our findings cannot yet explain the cultural or societal factors that influence these variations in prevalence. To illustrate, access to weapons and cultural norms surrounding their use may affect the prevalence of weapon carrying. Higher rates observed in the USA and the FYR Macedonia could be attributable to the availability of firearms and other weapons [54], while rates of weapon carrying in countries (e.g., Israel or FYR Macedonia) with a strong military presence or ongoing conflict may be attributable to differential societal norms in the face of ongoing societal conflict. Among girls, there are obvious variations in the prevalence of weapon carrying. Some of these may be attributable to weapons carried for defensive purposes as is common in many settings.

Findings suggested that both weapon carrying and physical fighting were each positively associated with reports of both physical and emotional health problems. This finding was expected, and demonstrates that both indicators are risk factors for adverse health outcomes. This finding was observed by country, gender, for each of the two health outcomes, and remained after simultaneous adjustment for multiple potential confounders. Second, we also observed geographic variations in these relationships, which indicate that context as indicated by country of origin does play a role in this process.

Study findings were consistent with a Problem Behaviour Model proposed by Jessor [12], [21], [22], [23]. The Problem Behaviour model suggests that behaviours represent a constellation of symptoms for a troubled adolescent which contribute to a trajectory of poor health outcomes. Children engaged in multiple risk behaviours may represent subgroups who are distressed [1], or feeling alienated or lacking support from adults and the rest of society [55], [56], [57]. Physical fighting and weapon carrying should therefore be viewed as part of this wider constellation of risk behaviours that co-occur and have important health consequences when viewed collectively.

Findings also showed that some behaviours with low frequencies in certain countries (e.g. physical fighting among girls in Israel or weapon carrying among girls in the FYR Macedonia) had high odds ratios for injuries and emotional health outcomes, while some behaviours with higher frequencies (e.g. weapon carrying in the US among boys and physical fighting among boys in Belgium) were associated with lower odds ratios of injuries and emotional health outcomes. In other words, in countries in which violence may be more common, it may prove to be less of a risk factor for health outcomes. These findings may be understood through the paradigm of the "normalization thesis" [58], [59] which suggests that the "risky" or deviant nature of risk behaviours needs to be assessed in a cultural context. "Normalization" refers to the situation in which risk behaviour has or is in the process of entering mainstream youth culture, attracting ordinary, and well-adjusted as opposed to deviant youth. Although the normalization thesis has been used to examine substance use, we would like to suggest that it may be a useful framework within which to examine cross-cultural differences in the relationship between violence and other risk behaviours, such that the more "normative" violence may be in a society, the less it will act as a risk factor.

Strengths and limitations of this study warrant recognition. The WHO-HBSC is a large survey involving many countries and a standard international protocol. This standardization of items and methodology makes it possible to perform robust analyses that examine the consistency of postulated health relationships in populations of young people in Europe, North America and the Middle East. Our analysis was large, adequately powered, novel, and focused upon a health issue of contemporary importance to most populations of young people. Limitations of the WHO-HBSC survey include its cross-sectional nature. While the evidence derived from our analyses satisfies most of the modern epidemiological criteria for causation (e.g., strength of statistical significance of associations, consistency, plausibility, biological gradients [60], other disciplines would demand that the temporal sequence of events be confirmed via longitudinal analyses. WHO-HBSC is also limited in terms of measures that explain the cultural factors that influence the occurrence of violence, and the need for youth to engage in such behaviours. Hence, conditions in each country that are impacting on levels of violence remain speculative.

Our results have implications for preventive intervention. Since our findings were supportive of a problem behaviour phenomenon, this study highlights the fact that fighting and weapon carrying are indicators of both physical and psychological health problems. Hence, routine assessment of the involvement of young people for these behaviours, especially in combination with other risk-taking behaviours, indicates a need for intervention to prevent future health problems. Our findings identify population subgroups, both demographic and social, that are particularly vulnerable and require targeted and perhaps tailored clinical and public health interventions. Known efficacious strategies include family-based training [61], school-based strategies involving individual counseling of violent children [62], the tailoring of interventions to racial or ethnic compositions of communities, with specific attention to family and community influences [63], as well as more general development of social skills and appropriate conflict resolution [64]. Such strategies could impact upon violence and associated problem behaviors.

In conclusion, this study explored the associations between fighting and weapon carrying and physical and emotional health outcomes. Results highlight the strong consistent relationship between fighting and weapon carrying and health risks, the impact of social and demographic context (as expressed by country of residence) and suggest the relevance of culture related normalized behaviour [58]. We conclude that youth violence is part of a constellation of risk behaviours with health implications, as opposed to being a distinct individual risk factor for impaired health in youth populations.
